# Supplementary material for: Functional role of Tet-mediated RNA hydroxymethylcytosine in mouse ES cells and during differentiation
Source: Nat Commun. 2020 Oct 2;11:4956. doi: 10.1038/s41467-020-18729-6 (PMC7532169; doi:10.1038/s41467-020-18729-6)
Supplement: Supplementary file 1 — Supplementary Information [file 41467_2020_18729_MOESM1_ESM.pdf]

Supplementary Information

Functional role of Tet-mediated RNA hydroxymethylcytosine in mouse ES cells and during differentiation

Jie Lan et al., Nature Communications 2020

Contents

Supplementary Figures 2

Supplementary Fig. 1 (related to Fig. 1): 6

Supplementary Fig. 2 (related to Fig. 2): 10

Supplementary Fig. 3 (related to Fig. 3): 14

Supplementary Fig. 4 (related to Fig. 5): 15

Supplementary Fig. 5 (related to Fig. 6): 17

Supplementary References 18

Supplementary Figures

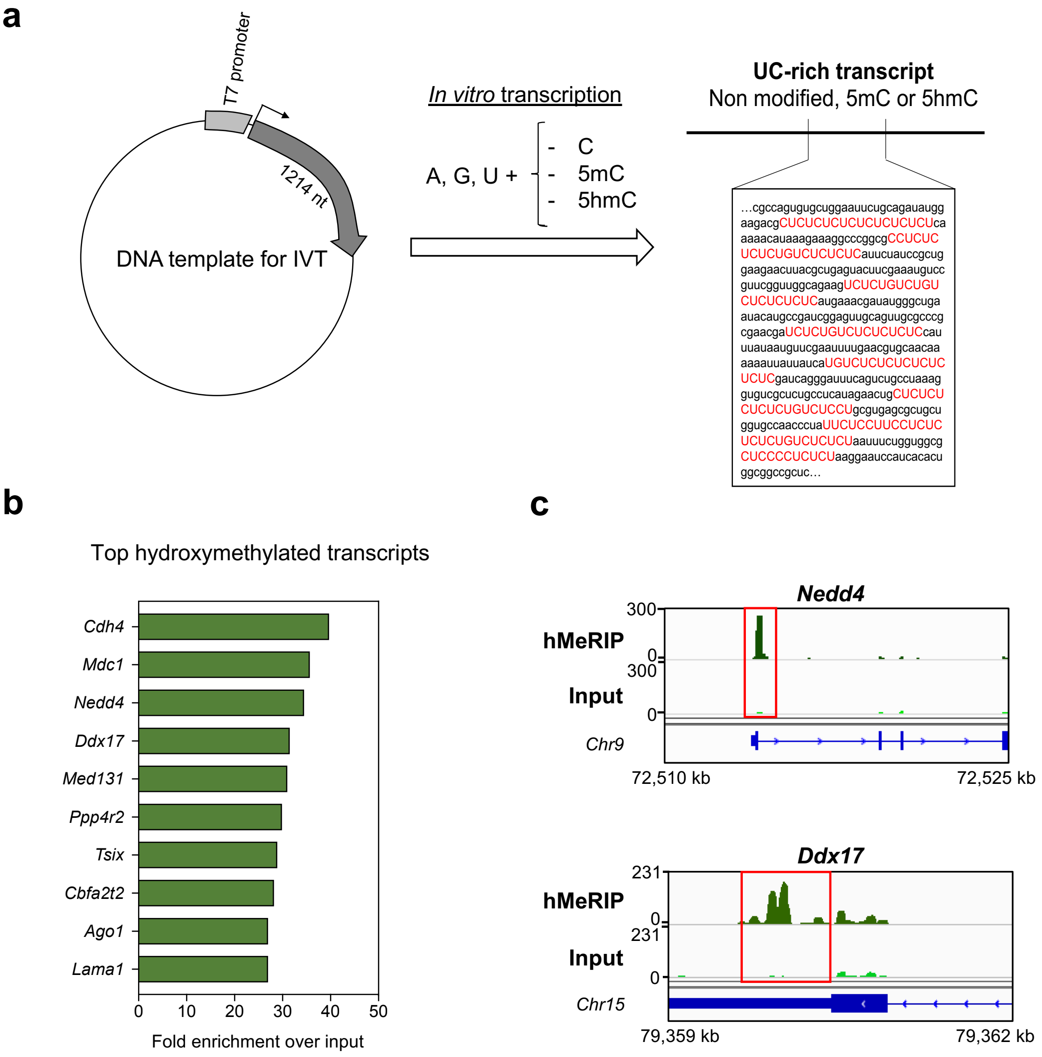

d

Rank	Motif	Position of best site in sequence	E-value
1	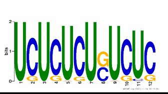	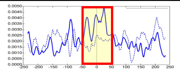 Center	2.2 x 10^{-117}
2	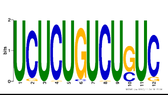	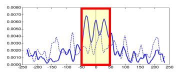 Center	4.0 x 10^{-123}
3	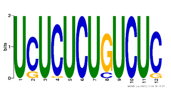	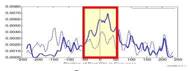 Center	7.9 x 10^{-116}

Supplementary Fig. 1a-d, Lan *et al.*

e

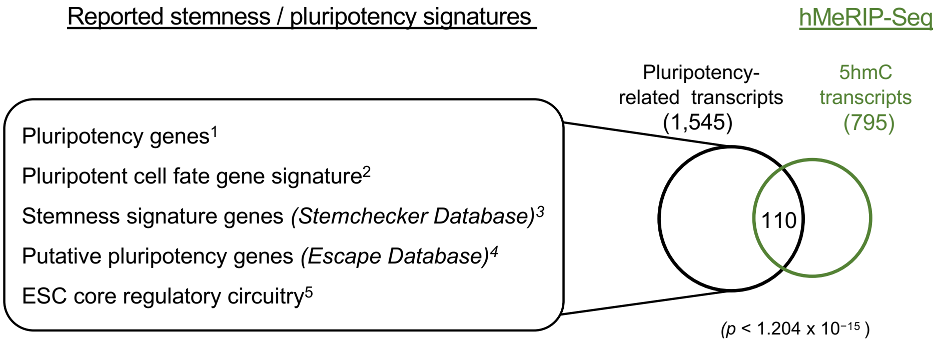

f

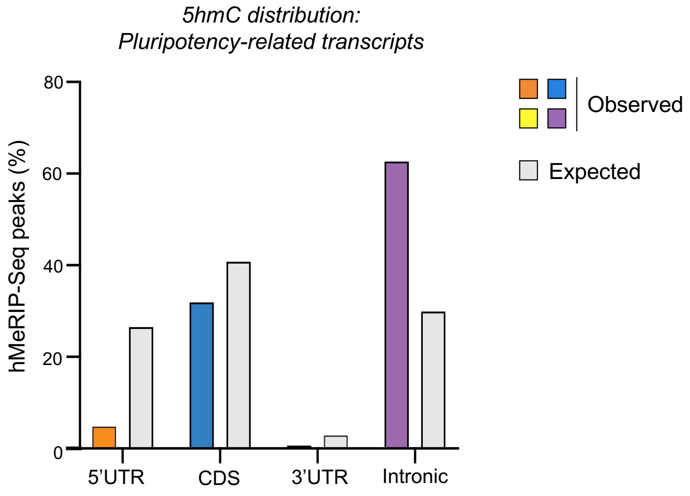

Supplementary Fig. 1e-f, Lan *et al.*

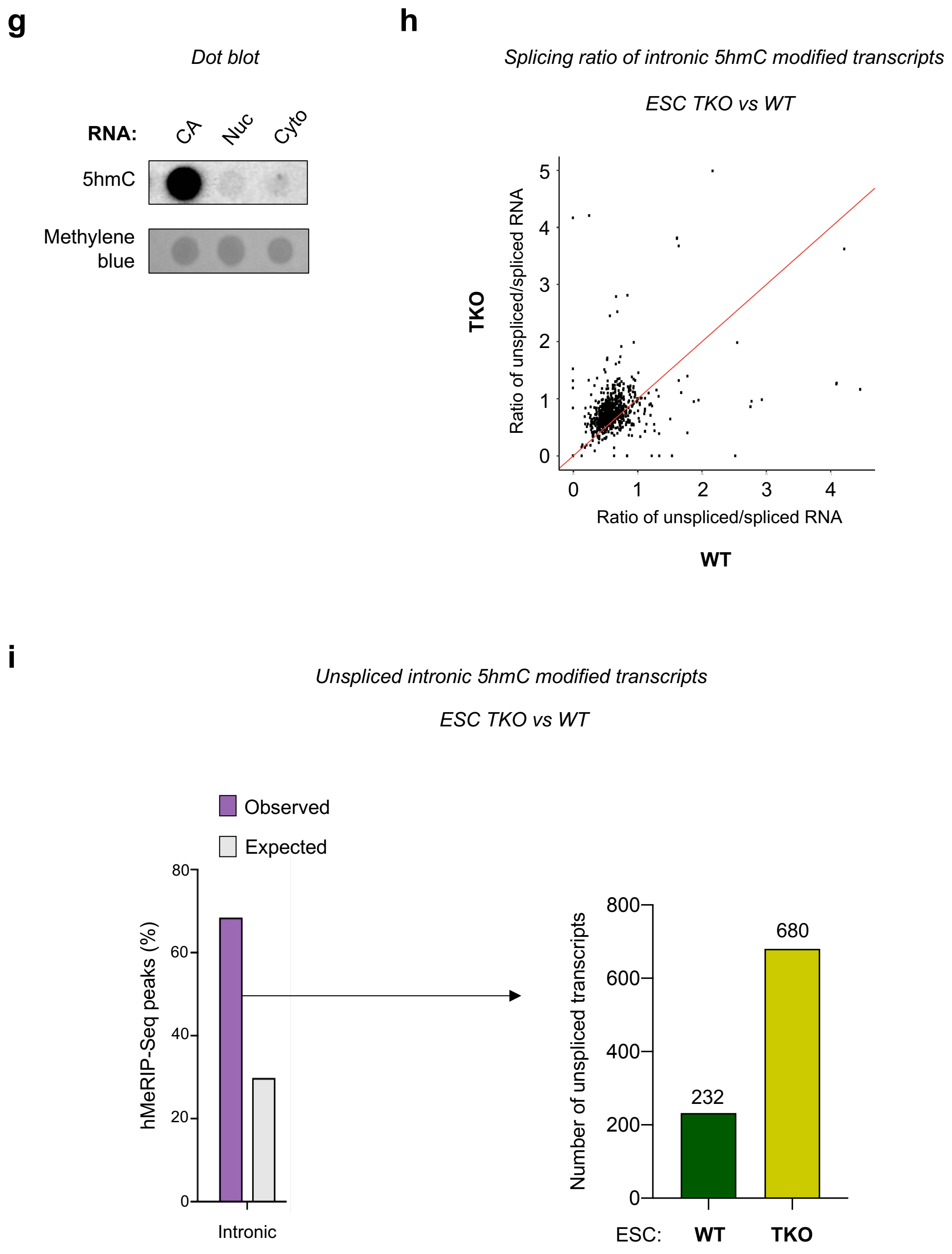

Supplementary Fig. 1g-i, Lan *et al.*

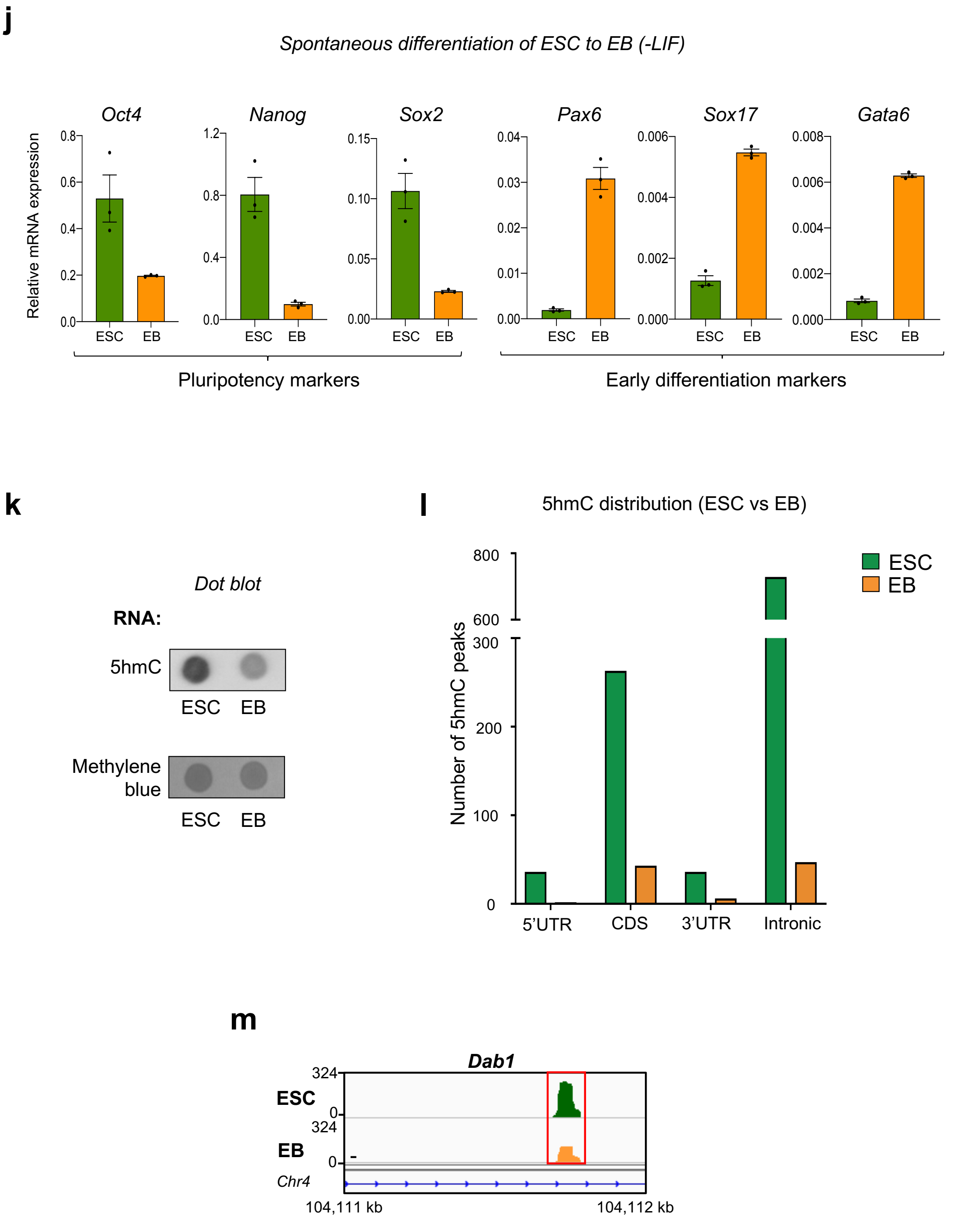

Supplementary Fig. 1j-m, Lan *et al.*

Supplementary Fig. 1 (related to Fig. 1):

a, Scheme illustrating the protocol of *in vitro* transcription (IVT). The plasmid used for IVT comprises a TC-rich cDNA harbouring all the motifs found by hMeRIP-Seq (cf. Fig. 1e and Supplementary Fig. 1d), flanked by a T7-polymerase-responsive promoter. The IVT experiment was performed in the presence of C, 5mC, or 5hmC nucleotides. **b,** Transcripts with the most significant 5hmC peaks (top 10), with their associated gene symbols and fold enrichment over input. **c,** Additional examples of hMeRIP-Seq profiles: transcripts of *Nedd4* and *Ddx17* in WT ESCs. **d,** Top sequence motifs identified at centres of 5hmC peaks. **e,** Scheme illustrating databases[1–5] of pluripotency-related transcripts and Venn diagram showing the overlap of pluripotency-related transcript datasets with 5hmC-modified transcripts. (Hypergeometric probability test). **f,** Bar chart showing the 5hmC peak distribution in pluripotency-related RNAs according to the type of structural element in the transcripts, next to the expected percentage. **g,** Dot blotting with 5hmC antibody after cell fractionation shows a high level of 5hmC in chromatin-associated RNA (CA), as opposed to nucleoplasmic RNA (Nuc) and cytoplasmic RNA (Cyto). A representative blot and its methylene blue control are shown (n=3 independent experiments). **h,** WT ESCs show a higher ratio of spliced to unspliced transcripts than TKO ESCs, for the ones bearing 5hmC in introns. Scatter plot showing the ratio of unspliced/spliced transcripts in TKO ESCs (y-axis) plotted against the ratio of unspliced/spliced transcripts in WT ESCs (x-axis). Every dot represents a single transcript. **i,** Bar chart showing the number of unspliced transcripts in WT vs TKO ESCs (right), among those bearing 5hmC in intronic regions obtained by hMeRIP-Seq (left). **j,** RT-qPCR analysis of transcripts encoding pluripotency markers and early differentiation markers, showing proper ESC-to-EB spontaneous differentiation. Data are means ± SEM (n=3 independent experiments). **k,** Dot blot assay of the 5hmC content of total RNA, showing substantial reduction of 5hmC in EBs as compared to ESCs (Representative

blots from three independent experiments). Methylene blue staining showing the loading control. **l,** Bar chart showing the difference in 5hmC peak distribution, between ESCs and EBs, among types of structural elements within transcripts. **m,** Additional example of hMeRIP-Seq profiles: profiles of *Dab1* transcripts, showing reduced 5hmC in EBs compared to ESCs (IGV tracks) (red frame shows peak location). Source data are provided as a Source Data File.

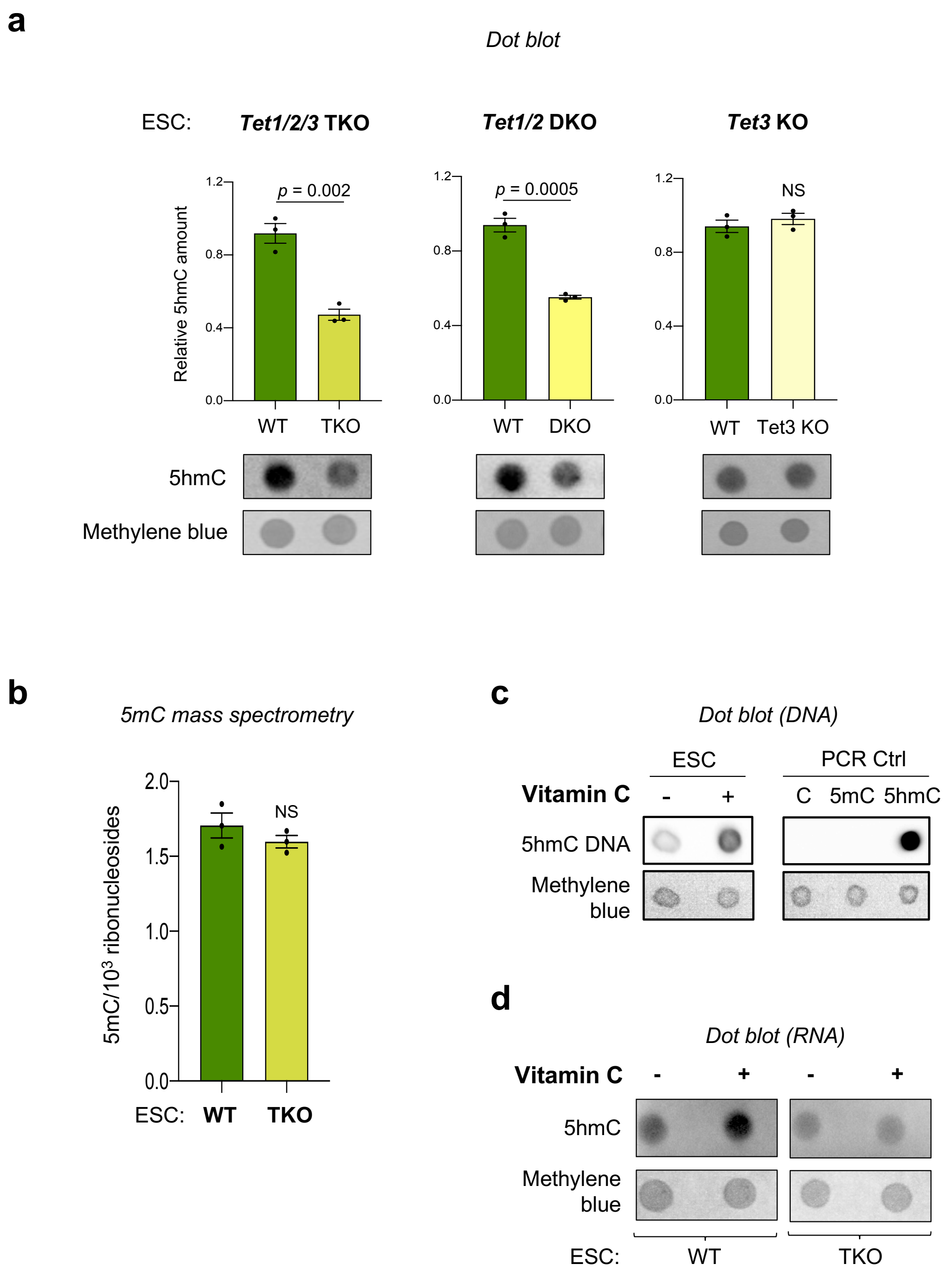

Supplementary Fig. 2a-d, Lan *et al.*

e

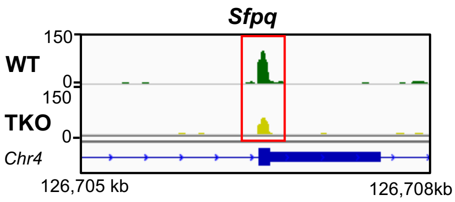

f

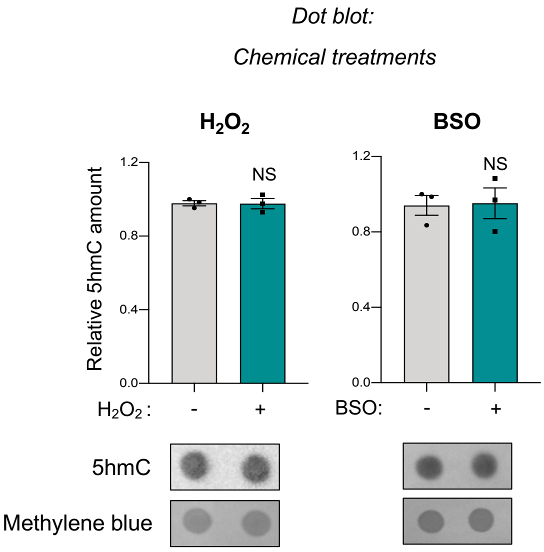

Supplementary Fig. 2e-f, Lan *et al.*

Supplementary Fig. 2 (related to Fig. 2):

a, Reduced global 5hmC level in Tet1/2/3 TKO and Tet1/2 DKO but not in Tet3 KO ESCs, as measured by dot blotting performed with total RNA and 5hmC antibody. Data are means ± SEM (n=3 independent experiments, one-tailed Student's *t*-test; NS, not significant). Shown with a representative blot and its methylene blue control. **b,** 5mC levels in WT and TKO ESCs, as measured by mass spectrometry. Data are means ± SEM (n=3 independent experiments, one-tailed Student's *t*-test, NS, not significative). **c,** Left: Control dot blot assay of the 5hmC content of DNA, showing a substantial increase in 5hmC in vitamin-C-treated as compared to untreated WT ESCs. Right: Control performed with unmarked, 5mC-marked, and 5hmC-marked PCR products, showing the specificity of the 5hmC antibody for 5hmC (Representative blots from three independent experiments). Methylene blue staining showing the loading control. **d,** Dot blot assay of the 5hmC content of total RNA, showing a substantial increase in 5hmC in vitamin-C-treated vs untreated WT ESCs, but no change in 5hmC in vitamin-C-treated vs untreated TKO ESCs (Representative blots from three independent experiments). Methylene blue staining showing the loading control. **e,** Additional exemplative hMeRIP-Seq profiles: the peak corresponding to *Sfpq* transcripts is reduced in TKO vs WT ESCs (IGV tracks) (red frame shows peak location). **f,** Absence of any global change in the 5hmC level as measured by dot blotting with total RNA and 5hmC antibody after treatment of ESCs with H_2O_2 or BSO, as compared to the corresponding vehicle-treated control. Data are means ± SEM (n=3 independent experiments, one-tailed Student's *t*-test; NS, not significant). Shown with a representative blot and its methylene blue control. Source data are provided as a Source Data File.

a

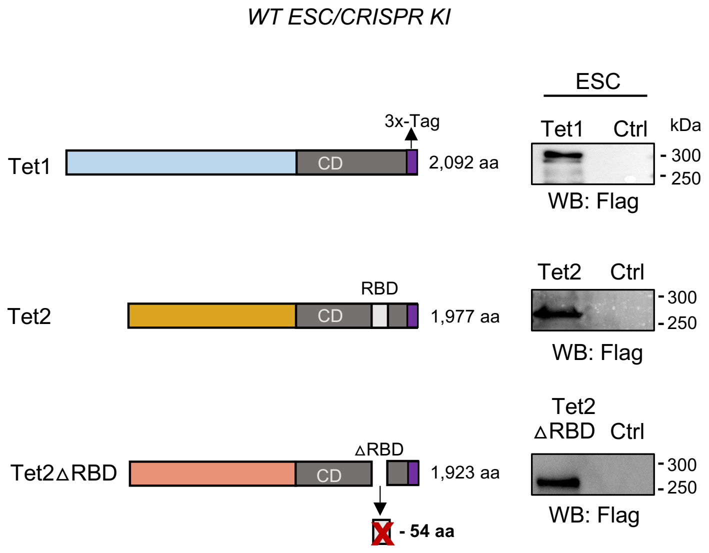

b

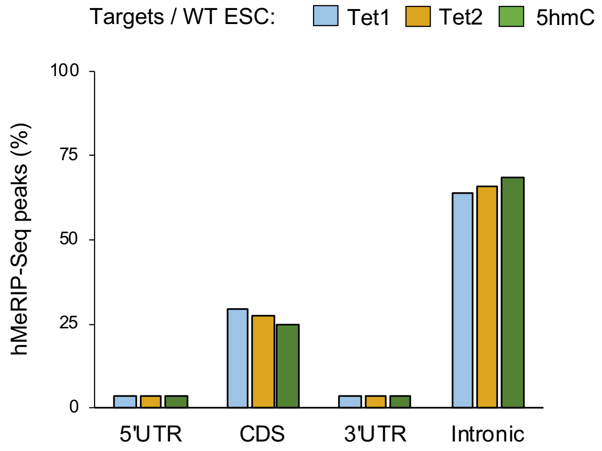

Supplementary Fig. 3a-b, Lan *et al.*

c

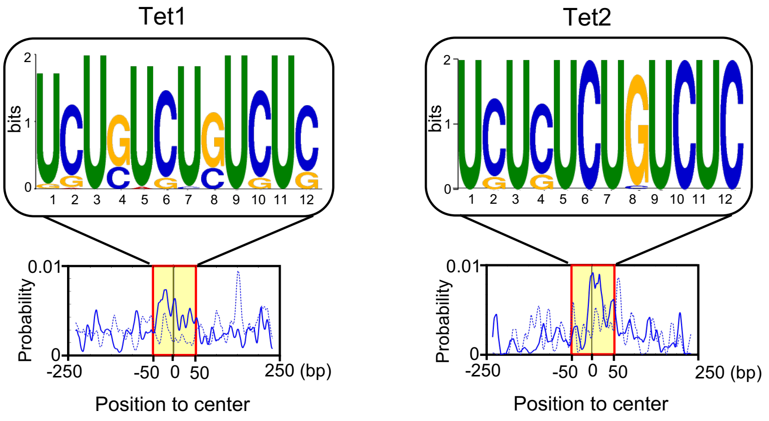

d

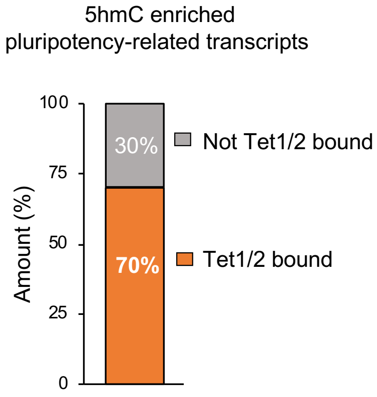

e

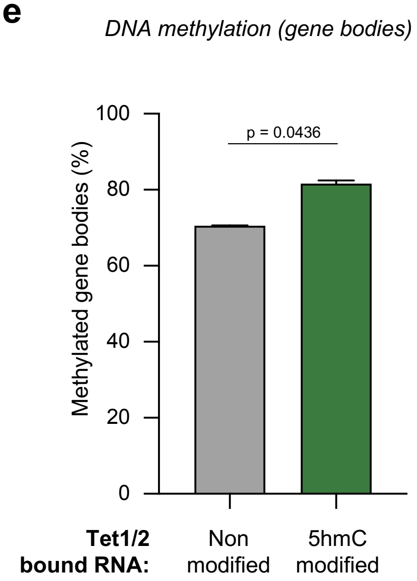

Supplementary Fig. 3c-e, Lan *et al.*

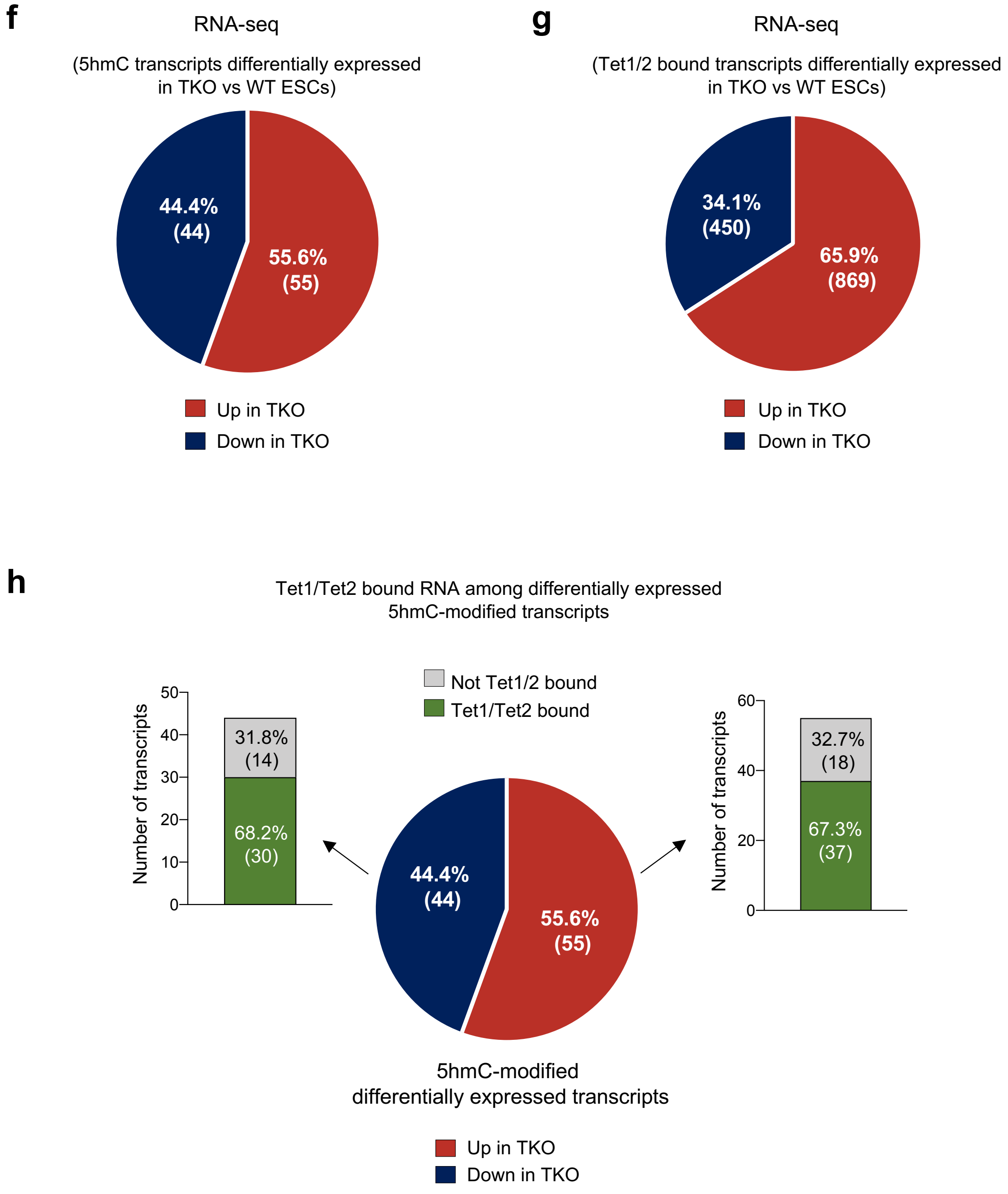

Supplementary Fig. 3f-h, Lan *et al.*

Supplementary Fig. 3 (related to Fig. 3):

a, Diagram illustrating CRISPR-mediated tagging of endogenous Tet1, Tet2, and Tet2ΔRBD proteins (Left). Western blots depict levels of tagged Tet1, Tet2, and Tet2ΔRBD proteins. For each, a representative blot from three independent experiment are shown. (right). **b,** Bar chart showing the distribution of 5hmC peaks, Tet1-binding sites, and Tet2-binding sites according to the type of structural element within the transcript. **c,** Top sequence motifs identified at Tet1- and Tet2 binding sites within 5hmC-enriched targets (E-values: 2.0e-067 and 7.7e-062, respectively). **d,** Stacked bar chart showing the overlap between the 110 5hmC-modified pluripotency-related transcripts and RNA targets of Tet1 and/or Tet2. **e,** Bar chart showing the percentage of methylated gene bodies whose transcripts are bound by Tet1 and/or Tet2, and are not modified or 5hmC modified using published data[6]. Data are means ± SEM (Two-tailed Student t-test). **f,** Pie chart showing the percentage of 5hmC-marked transcripts differentially expressed in TKO compared to WT ESCs. **g,** Pie chart showing the percentage of Tet1/2-bound transcripts differentially expressed in TKO compared to WT ESCs. **h,** Pie chart showing the percentage of 5hmC-marked transcripts differentially expressed in TKO compared to WT ESCs, with a related stacked bar chart showing the percentage of Tet1/2-bound transcripts among the differentially regulated ones.

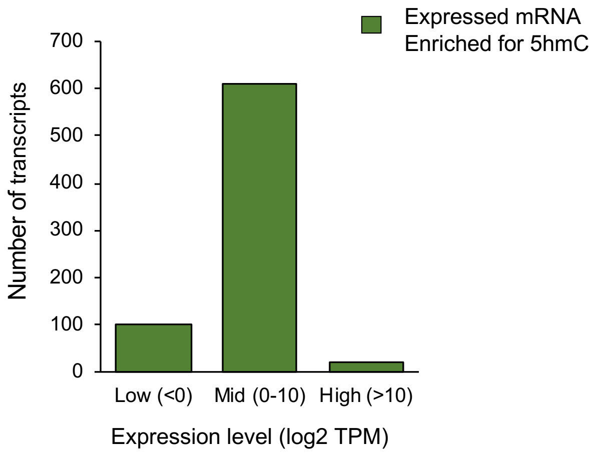

Supplementary Fig. 4, Lan *et al.*

Supplementary Fig. 4 (related to Fig. 5):

Bar chart showing in ESCs the distribution of identified 5hmC-modified transcripts (green) according to their abundance (high, medium, or low expression of the corresponding gene; TPM, transcripts per million).

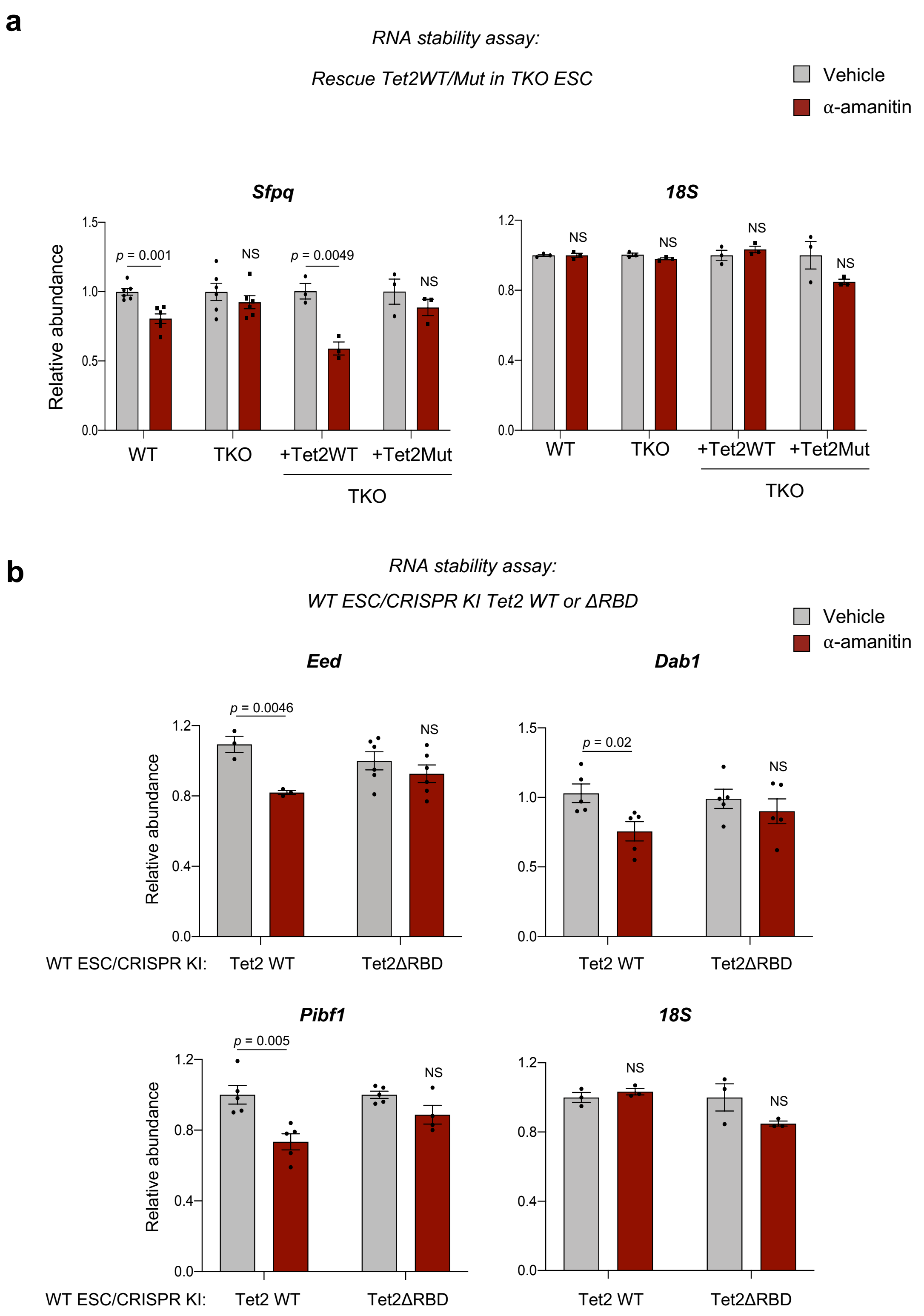

Supplementary Fig. 5, Lan *et al.*

Supplementary Fig. 5 (related to Fig. 6):

a, Bar graphs showing relative abundances of *Sfpq* transcript and control *18S* rRNA after transcriptional inhibition with α-amanitin in WT, TKO, TKO + Tet2WT, and TKO + Tet2Mut cells, as compared to control cells treated with vehicle. *18S* rRNA was used as an internal calibrator. Data are means ± SEM for at least 3 independent experiments. (Two-tailed Student's *t*-test; NS, not significant). **b,** 5hmC-modified transcripts show higher stability in ESCs producing Tet2ΔRBD than in control cells producing Tet2 WT. Bar graphs showing relative abundances of *Eed*, *Dab1*, *Pibf1* and *18S* transcripts in Tet2 WT and Tet2ΔRBD ESCs treated with α-amanitin (for transcriptional inhibition) as compared to control cells treated with vehicle. *18S* rRNA was used as an internal calibrator. Error bars indicate ± SEM for at least 3 independent experiments. (Two-tailed Student's *t*-test; NS, not significant). Source data are provided as a Source Data File.

Supplementary References

1. Geula, S. *et al.* Stem cells. m6A mRNA methylation facilitates resolution of naïve pluripotency toward differentiation. *Science* **347**, 1002–1006 (2015).
2. Fidalgo, M. *et al.* Zfp281 Coordinates Opposing Functions of Tet1 and Tet2 in Pluripotent States. *Cell Stem Cell* **19**, 355–369 (2016).
3. Pinto, J. P. *et al.* StemChecker: a web-based tool to discover and explore stemness signatures in gene sets. *Nucleic Acids Res.* **43**, W72-77 (2015).
4. Xu, H. *et al.* ESCAPE: database for integrating high-content published data collected from human and mouse embryonic stem cells. *Database* **2013**, (2013).
5. Young, R. A. Control of the embryonic stem cell state. *Cell* **144**, 940–954 (2011).
6. Ficz, G. *et al.* Dynamic regulation of 5-hydroxymethylcytosine in mouse ES cells and during differentiation. *Nature* **473**, 398–402 (2011).
