# Supplementary material for: Functional role of Tet-mediated RNA hydroxymethylcytosine in mouse ES cells and during differentiation
Source: Nat Commun. 2020 Oct 2;11:4956. doi: 10.1038/s41467-020-18729-6 (PMC7532169; doi:10.1038/s41467-020-18729-6)
Supplement: Supplementary file 3 — Reporting Summary [file 41467_2020_18729_MOESM3_ESM.pdf]

nature research

Corresponding author(s): François Fuks
Last updated by author(s): Jul 17, 2020

Reporting Summary

Nature Research wishes to improve the reproducibility of the work that we publish. This form provides structure for consistency and transparency in reporting. For further information on Nature Research policies, see Authors & Referees and the Editorial Policy Checklist .

Statistics

For all statistical analyses, confirm that the following items are present in the figure legend, table legend, main text, or Methods section.

n/a	Confirmed	
☐	☒	The exact sample size (*n*) for each experimental group/condition, given as a discrete number and unit of measurement
☐	☒	A statement on whether measurements were taken from distinct samples or whether the same sample was measured repeatedly
☐	☒	The statistical test(s) used AND whether they are one- or two-sided<br>*Only common tests should be described solely by name; describe more complex techniques in the Methods section.*
☒	☐	A description of all covariates tested
☒	☐	A description of any assumptions or corrections, such as tests of normality and adjustment for multiple comparisons
☐	☒	A full description of the statistical parameters including central tendency (e.g. means) or other basic estimates (e.g. regression coefficient) AND variation (e.g. standard deviation) or associated estimates of uncertainty (e.g. confidence intervals)
☐	☒	For null hypothesis testing, the test statistic (e.g. *F*, *t*, *r*) with confidence intervals, effect sizes, degrees of freedom and *P* value noted<br>*Give P values as exact values whenever suitable.*
☒	☐	For Bayesian analysis, information on the choice of priors and Markov chain Monte Carlo settings
☒	☐	For hierarchical and complex designs, identification of the appropriate level for tests and full reporting of outcomes
☒	☐	Estimates of effect sizes (e.g. Cohen's *d*, Pearson's *r*), indicating how they were calculated

Our web collection on statistics for biologists contains articles on many of the points above.

Software and code

Policy information about availability of computer code

Data collection: qPCR data was acquired on the LightCycler480 real-time PCR system (Roche). Western blots and dot blots were developed with the ECL system (Amersham Biosciences). Mass spectrometry data was collected by an AB 3200 QTRAP mass spectrometer (Applied Biosystems, Foster City, CA, USA). hMeRIP-Seq, RIP-Seq, and RNA-Seq data were collected by NextSeq500 system (Illumina).

Data analysis

- Graphpad prism 7 and the computing environment R v3.2.0 were used for statistics.
- ImageJ software was used for signal quantification.
- The raw sequencing data was analysed with FastQC v0.11.4.
- Low-complexity reads were removed with the AfterQC tool v0.9.1.
- Reads derived from tRNA or rRNA were mapped to mouse tRNA and rRNA sequences with Bowtie2 v2.2.5.
- Adapters on reads were processed with Trimmomatic v0.36.
- Preprocessed reads were mapped against the mouse reference genome (mm9) with either the STAR v2.5.1b (RNA-Seq and hMeRIP-Seq), RSEM v1.2.31 (RIP-Seq) or Bowtie2 v2.2.5 (MeDIP-Seq).
- Duplicated mapped reads from MeDIP were filtered using picard v2.20.4.
- Peak regions were identified by applying the MACS2 peak-calling tool v2.1.0.20150731.
- Bedtools v2.25.0 was used to compute peaks overlap and extract peak sequences.
- FeatureCounts v1.5.1. was used to count reads covering peaks and derived enrichment levels.
- Peaks were visualized in IGV tool v2.3.82(130).
- Motif analysis was performed with the meme-suite v4.11.1.
- Gene expression was computed with the HTseq tool v0.6.1p1.
- Differential analysis was performed with the DESeq2 tool v3.11.
- Splicing analysis was performed with IR Finder v1.2.3.
- Code supporting this study are available at a dedicated Github repository [https://github.com/martinBizet/hmC_ES].

For manuscripts utilizing custom algorithms or software that are central to the research but not yet described in published literature, software must be made available to editors/reviewers. We strongly encourage code deposition in a community repository (e.g. GitHub). See the Nature Research guidelines for submitting code & software for further information.

Data

Policy information about availability of data

All manuscripts must include a data availability statement. This statement should provide the following information, where applicable:

- Accession codes, unique identifiers, or web links for publicly available datasets
- A list of figures that have associated raw data
- A description of any restrictions on data availability

- All sequencing data that support the findings of this study have been deposited in NCBI's Gene Expression Omnibus(GEO) under the accession code: GSE131902.
- Stemness/pluripotency signature genes was derived from the ESCAPE database (http://www.maayanlab.net/ESCAPE/), the StemChecker database (http://stemchecker.sysbiolab.eu/), and from published data (Geula et al Science 2015; Fidalgo et al Cell Stem Cell 2016; Young Cell 2011).
- Published microarray data for mRNA half-lifes were obtained from Sharova et al DNA Res 2009 (DOI: 10.1093/dnares/dsn030) (Supplementary table 1).
- Published Ribo-Seq data were obtained from Ingolia et al Cell 2011 (DOI: 10.1016/j.cell.2011.10.002) (Supplementary table 1).
- Published MeDIP-Seq were obtained from Ficz et al Nature 2011 (Accession code: ERP000570).
- The source data underlying Figs 1a, g, h; 2a; 6b, d and Supplementary Figs 1g, j, k; 2a-d, f; 3a and 5a-b are provided as a Source Data file.

Field-specific reporting

Please select the one below that is the best fit for your research. If you are not sure, read the appropriate sections before making your selection.

☒ Life sciences ☐ Behavioural & social sciences ☐ Ecological, evolutionary & environmental sciences

For a reference copy of the document with all sections, see nature.com/documents/nr-reporting-summary-flat.pdf

Life sciences study design

All studies must disclose on these points even when the disclosure is negative.

Sample size	Sample size, number of replicates, errors bars and statistical tests were chosen based on experience and variability of in vitro studies, and stated in each figure legend. Unless otherwise indicated, all experiments included technical replicates and were repeated at least three independent times. No statistical methods were used to predetermine sample sizes. No in vivo studies were performed.
Data exclusions	No data were excluded from analysis.
Replication	All attempts to replicate experiments were successful. Details on replication of each particular experiments are provided in Figure Legends.
Randomization	For each experiment, a subpopulation of cells was allocated randomly from the whole population of cells with clear genotype without using specific randomization methods.
Blinding	No blinding was applied in this study, as all data produced derived from objective quantitative methods.

Reporting for specific materials, systems and methods

We require information from authors about some types of materials, experimental systems and methods used in many studies. Here, indicate whether each material, system or method listed is relevant to your study. If you are not sure if a list item applies to your research, read the appropriate section before selecting a response.

Materials & experimental systems

n/a	Involved in the study
☐	☒ Antibodies
☐	☒ Eukaryotic cell lines
☒	☐ Palaeontology
☒	☐ Animals and other organisms
☒	☐ Human research participants
☒	☐ Clinical data

Methods

n/a	Involved in the study
☒	☐ ChIP-seq
☒	☐ Flow cytometry
☒	☐ MRI-based neuroimaging

Antibodies

Antibodies used

- Mouse monoclonal anti-Flag Tag (Sigma, #F1804) for western blots and RIP-Seq.
- Rat monoclonal anti-5hmC (Diagenode, #MAb-633HMC) for dot blots and hMeRIP-Seq.
- Rabbit polyclonal anti-Rat IgG HRP (Abcam #Ab6734) for dot blots.
- Amersham ECL Rabbit IgG, HRP-linked whole Ab (from donkey) (Sigma, GE Healthcare, #NA934-1ML) for western blots.
- Amersham ECL Mouse IgG HRP-linked whole Ab (from sheep) (Sigma, GE Healthcare, #NA931-1ML) for western blots.

Validation

All antibodies used are commercially available. Based on the information from manufacturer's websites, all primary antibodies are validated. Anti-Flag Tag (Sigma, F1804) provides a single band of protein on a Western Blot from mammalian crude cell lysates by chemiluminescent probing and is recommended for immunoblotting, immunoprecipitation, immunohistochemistry, immunofluorescence and immunocytochemistry. Besides, this antibody has been extensively used in previous peer-reviewed publications (>3000). Anti-5hmC (Diagenode, #MAb-633HMC) has been extensively validated by the manufacturer through hMeRIP assays, and dot blot analysis with C, 5mC and 5hmC PCR controls, and has been further validated in our study (Fig. 1a) and in our previous publication (Delatte et al Science 2016)

Eukaryotic cell lines

Policy information about cell lines

Cell line source(s)

The mouse wild type, Tet1/2/3 triple knockout (TKO) and Tet1/2 double knockout (DKO) ESCs used in this study were obtained from Prof. Rudolf Jaenisch (Massachusetts Institute of Technology, Cambridge, USA).
The mouse Tet3 knockout ESCs used in this study were obtained from Prof. Anjana Rao (University of California, USA).
Mouse ESC lines producing tagged- Tet1,-Tet2, and -Tet2ΔRBD are generated in-house via CRISPR-Cas9 nickase system.

Authentication

The cell lines used were not authenticated.

Mycoplasma contamination

All cell lines were tested negative for mycoplasma contamination.

Commonly misidentified lines
(See ICLAC register)

No cell lines used in this study were found in the database of commonly misidentified cell lines that is maintained by ICLAC.
