# Supplementary material for: Functional role of Tet-mediated RNA hydroxymethylcytosine in mouse ES cells and during differentiation
Source: Nat Commun. 2020 Oct 2;11:4956. doi: 10.1038/s41467-020-18729-6 (PMC7532169; doi:10.1038/s41467-020-18729-6)
Supplement: Supplementary file 4 — Description of Additional Supplementary Files [file 41467_2020_18729_MOESM4_ESM.pdf]

Description of Additional Supplementary Files

Supplementary Data 1. List of enriched hMeRIP-Seq peaks in WT ESC.

Supplementary Data 2. List of ratio of spliced to unspliced transcripts bearing intronic 5hmC in WT ESC and TKO ESC.

Supplementary Data 3. List of differential hMeRIP-Seq peaks between WT ESC and EB.

Supplementary Data 4. List of differential hMeRIP-Seq peaks between WT ESC and TKO ESC.

Supplementary Data 5. List of enriched RIP-Seq transcripts in tagged-Tet1, -Tet2 and -Tet2ΔRBD ESCs.

Supplementary Data 6. List of differential hMeRIP-Seq peaks between tagged-Tet2 WT and -Tet2ΔRBD ESCs.

Supplementary Data 7. List of differentially expressed mRNAs between WT ESC and TKO ESC identified by RNA-Seq after transcription inhibition.

Supplementary Data 8. List of primers and oligos used for RT-qPCR, genotyping, Crispr-gRNA and plasmid cloning.
